# Better quality of life and less caregiver strain in young-onset Parkinson’s disease: a multicentre retrospective cohort study

**DOI:** 10.1007/s00415-020-10266-y

**Published:** 2020-10-27

**Authors:** Maarten Te Groen, Bastiaan R. Bloem, Samuel S. Wu, Bart Post, The Parkinson’s Foundation Quality Improvement Initiative investigators

**Affiliations:** 1 Department of Neurology, Centre of Expertise for Parkinson and Movement Disorders, Donders Institute for Brain, Cognition and Behaviour, Radboud University Medical Centre, Geert Grooteplein Zuid 10, 6525GA Nijmegen, The Netherlands; 2 University of Florida, Miami, FL USA

**Keywords:** Parkinson’s, Disease, Young-onset, Quality of life, Caregivers

## Abstract

**Background:**

Parkinson’s disease (PD) is typically considered as a disease of the elderly. However, there is a sizeable subgroup of patients where PD starts at a younger age, known as young-onset PD (YOPD). We evaluated the differences in quality of life and caregiver strain between YOPD and later onset PD (LOPD) patients in a large cohort.

**Methods:**

In collaboration with the Parkinson Foundation Quality Improvement Initiative (PF-QII), we conducted a retrospective three-year analysis on 962 PD patients of the QII database (starting date May 2016). Of these, 272 patients had YOPD, and 690 had LOPD. The Parkinson’s Disease Questionnaire-39 (PDQ-39) total score served as primary outcome measure. Furthermore, we analysed group differences in modified caregiver strain index (MCSI) total score, three cognition functions, and number of falls. A regression analysis adjusting for covariates was used to assess the association of age at onset with PDQ-39 and MCSI.

**Results:**

PDQ scores were better in YOPD patients, MCSI scores on social constraint were lower in YOPD patients, but scores on financial constraint were higher in this group. After adjusting for covariates, YOPD patients had better quality of life and less caregiver strain at all follow-up moments, but not at baseline. Decline over time for all outcomes was lower in the YOPD group compared to the LOPD group. Cognitive functioning and number of falls progressed slower in the YOPD group compared to the LOPD group.

**Conclusion:**

Compared to LOPD patients, YOPD patients had a better quality of life, less caregiver strain, fewer falls and better cognitive functioning after their first follow-up visit, and also a slower decline over time.

**Electronic supplementary material:**

The online version of this article (10.1007/s00415-020-10266-y) contains supplementary material, which is available to authorized users.

## Introduction

Parkinson’s disease (PD) is associated with a great impact on quality of life of patients [1, 2]. Quality of life of PD patients is mainly dependent on disease severity and disability, as well as neuropsychiatric symptoms like depression [1–4]. Besides these symptoms, the gradual loss of autonomy leads to many alterations in the lives of patients and their spouses [5]. PD produces a progressive strain on spouses of patients when they become informal caregivers during disease progression [6, 7]. Earlier cross-sectional studies showed young-onset PD (YOPD) patients having poorer emotional well-being and lower quality of life [8, 9]. However, we hypothesize that YOPD patients, after being initially more struck by their diagnosis, have better capabilities to adapt to their disease and a stronger social network, resulting in better quality of life over time, as studies into other diseases with a marked impact on quality of life have pointed out [10–12]. We will specifically study the relation between quality of life, caregiver strain and YOPD, which thus far remains unclear. Insights into quality of life in relation with disease progression could improve care delivery and optimise the support for this often overlooked group of patients.

## Subjects and methods

We performed a cross-sectional and longitudinal retrospective analysis on 8334 PD patients of the Parkinson’s Foundation Quality Improvement Initiative (PF QII) database as of May 2016. All PD patients from the participating centers were eligible for inclusion. From this cohort, we selected all patients with an age at onset under 50 (YOPD) and those with age above 70, we called late-onset PD (LOPD). The PF QII study is one of the first large-scale studies describing quality of care amongst Parkinson’s disease patients seen in 24 international PF Centres of Excellence. Registry was done by administering a questionnaire by a qualified nurse, research coordinator or doctor, consisting of multiple categories including, amongst others, Parkinson’s Disease Questionnaire-39 (PDQ-39), Modified Caregiver Strain Index (MCSI), cognitive functioning, Timed Up and Go Test (TUGT), number of falls, type and number of medication [13]. All patients and caregivers signed informed consent before being admitted.

## Measures

Patients included in the PF QII-study completed a form, consisting of a section ‘patient data’, ‘patient diagnosis and PD stage’, ‘comorbid conditions’, ‘medication’, ‘other therapies’ and ‘clinical condition/outcomes’. A copy of the form can be found in the appendix. The primary outcome is PDQ-39 total score. Secondary outcomes are MCSI total score, cognitive functioning, and number of falls. The PDQ-39 is a 39-item validated scale completed by PD patients [14]. It contains eight dimensions, mobility, daily activities, emotional well-being, stigma, social support, cognition, communication and bodily discomfort. Out of each score for these dimensions one total score is calculated. It ranges from 0 to 100 where higher scores correlate with poorer Quality of Life. The MCSI is a questionnaire containing six dimensions of constraint, e.g., physical, social, financial, time, interpersonal strain and other demanding/manipulative behaviour. Scores range from 0 to 4 depending on the level of strain on caregivers. The scores for each domain are added up for a total score up to 72 points. Only this total score is used for analysis. Higher scores indicate more strain. The outcome cognition is based on standardized cognition tests, e.g., three cognition functions, immediate five-word recall, verbal fluency and delayed five-word recall. The number of falls is based on patient-derived information and categorized in ‘none/rarely’ and ‘at least monthly’.

### Data analysis

**Fig. 1:**
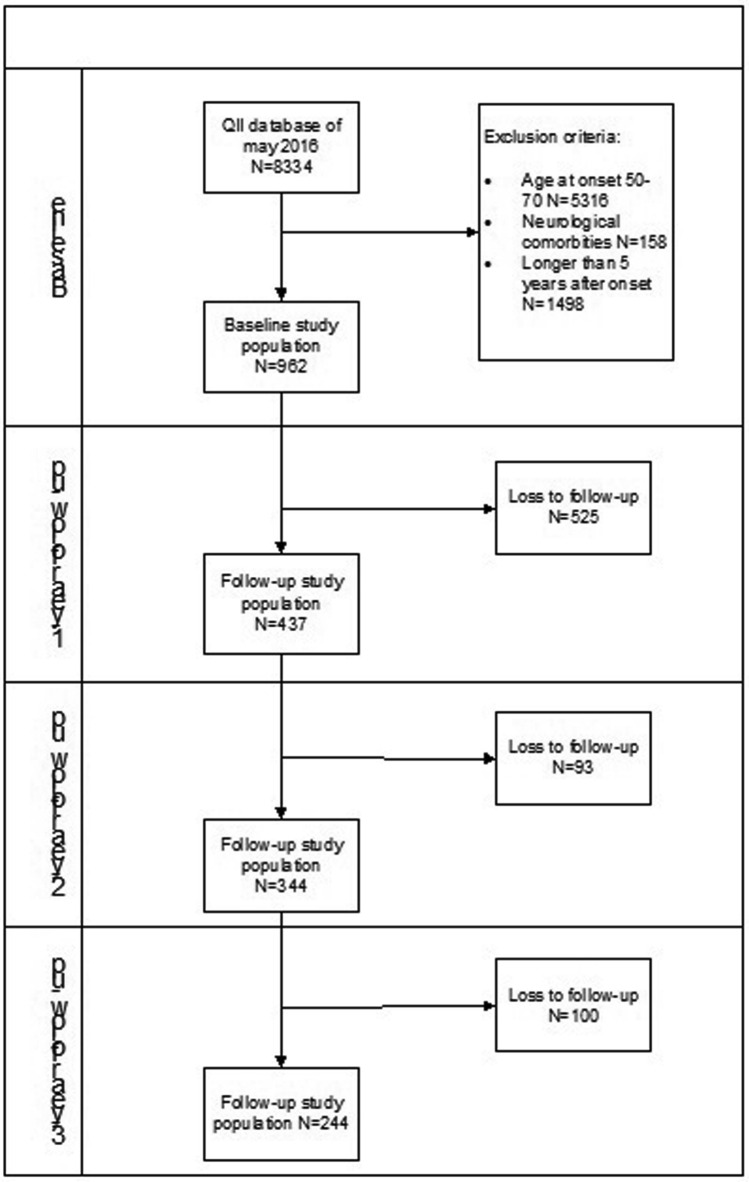
Study population flowchart. *QII* Qualitive Improvement Initiative

We performed a cross-sectional and longitudinal analysis on 8334 PD patients of the QII database as of May 2016. We excluded patients with missing age at onset, patients aged between 50 and 70 years old (*n* = 5316), with neurological comorbidities (*n* = 158) and patients with a disease duration longer than 5 years after onset (*n* = 1481). We included 272 patients with YOPD, and 690 patients with LOPD. For the longitudinal analysis, we included records at baseline, 1-, 2- and 3-years after follow-up. Total sample sizes at each year after baseline were *n* = 962, *n* = 437, *n* = 344 and *n* = 244 (Fig. 1).

Univariable data analysis was done using Student’s *t* tests. Multiple regression analysis was used for the cross-sectional as well as for the longitudinal data to adjust for disease duration, number of symptomatic co-morbidities, and number of medications used before baseline visit. A mixed model with subjects as random effects and unstructured covariance was used to calculate fixed effect estimates. The effect of each dependent variable on the outcome scores was calculated to show the relation between each other. Results for cognitive tests were presented as standardized z-scores.

## Results

### Descriptive statistics

YOPD patients had a higher weight (81.9 +  − 19.7 kg vs. 74.7 +  − 14.4 kg, *p* < 0.0001) without significant difference in BMI. They were more often men (67.6% vs. 60.7%, *p* = 0.046) and living at home (99.6% vs. 93.8%, *p* < 0.001). Regular care partners were more often spouses or relatives (80.5% vs. 67.2%, *p* < 0.001) and less often paid caregivers or other relatives (5.2% vs. 12.3%, *p* < 0.001).

LOPD patients had a slightly earlier objective (physician-based) assessment after onset of first subjective symptoms than YOPD patients (2.9 +  − 1.4 vs. 3.4 +  − 1.3 years, *p* < 0.0001). The Hoehn and Yahr (HY) stage of the LOPD group was higher compared to the YOPD group. There were significantly more symptomatic co-morbidities (excluding neurological co-morbidities) in the LOPD group (0.6 +  − 0.6 vs. 0.2 +  − 0.5, *p* < 0.0001).

There was no significant difference in falls. YOPD patients had a better TUG (10.2 +  − 5.6 vs. 16.2 +  − 8.9, *p* < 0.0001) and had higher cognitive function scores (*p* < 0.0001). The total PDQ-39 score was not significantly different between groups (19.9 +  − 15.2 vs. 21.0 +  − 14.3, *p* = 0.299), as was the total MCSI score (13.9 +  − 13.8 vs. 16.0 +  − 13.9, *p* = 0.137) (Table 1). However, looking at PDQ39 and MCSI sub-scores, YOPD patients had a significantly better mobility, ADL, emotional status, social support and cognition scores. Social constraints were higher, but financial constraints were lower in this group.

**Table 1:** Baseline characteristics

		Parkinson onset		*p* values
		YOPD (*n* = 272)	LOPD (*n* = 690)	
Age at first onset		46.3 ± 6.2	78.8 ± 4.5	** < 0.0001**
Age at onset		43.0 ± 6.1	75.9 ± 4.4	** < 0.0001**
Weight		81.9 ± 19.7	74.7 ± 14.4	** < 0.0001**
Gender (%male)		184 (67.6%)	419 (60.7%)	**0.046**
Living situation	At home	271 (99.6%)	645 (93.8%)	** < 0.001**
	Skilled care	1 (0.4%)	34 (4.9%)	
	Other	0 (0.0%)	9 (1.3%)	
Regular care partner	No	37 (13.6%)	135 (19.6%)	** < 0.001**
	Spouse/Partner	219 (80.5%)	463 (67.2%)	
	Other Relative	13 (4.8%)	56 (8.1%)	
	Paid Caregiver	1 (0.4%)	29 (4.2%)	
	Other	2 (0.7%)	6 (0.9%)	
Stand unaided	Yes	2 (0.7%)	49 (7.3%)	** < 0.0001**
	No	268 (99.3%)	622 (92.7%)	
#Of years from symptom to onset		3.4 ± 1.3	2.9 ± 1.4	** < 0.0001**
#Of years from diagnosis to assessment		2.0 ± 1.4	2.1 ± 1.4	0.710
H&Y stage	1	92 (35.8%)	89 (13.8%)	** < 0.0001**
	2	154 (59.9%)	340 (52.6%)	
	3	10 (3.9%)	173 (26.7%)	
	4–5	1 (0.4%)	45 (7.0%)	
#Of symptomatic comorbidities		0.2 ± 0.5	0.6 ± 0.9	** < 0.0001**
Falls	None/rare	225 (94.1%)	531 (90.3%)	0.074
	At least monthly	14 (5.9%)	57 (9.7%)	
PDQ-39 total		19.9 ± 15.2	21.0 ± 14.3	0.299
MCSI total		13.9 ± 13.8	16.0 ± 13.9	0.137

*YOPD* young-onset Parkinson’s disease, *LOPD* late-onset Parkinson’s disease, *H&Y* Hoehn and Yahr, *PDQ-39* Parkinson’s disease questionnaire 39, *MCSI* modified caregiver strain index

Values stated as mean +  − standard deviation or *n* (%)

The bold values indicate p < 0.05

YOPD patients were less often than LOPD patients treated with any form of Levodopa, and there was higher use of dopamine agonists, MAO-B inhibitors and amantadine, but less use of cognitive enhancers (*p* < 0.001). YOPD patients were less likely to be on other treatments before the first visit, with significant differences in physical, occupational and speech therapy, and with more patients having deep brain stimulation (supplementary table 1.).

### Cross-sectional results

Linear regression analyses unadjusted for covariates showed no significant differences on baseline for PDQ39, MCSI and falls. YOPD patients did perform better on cognitive functioning for all three tests (*p* < 0.001). After adjustment for disease duration, number of symptomatic co-morbidities and number of medications, there was an additional significant difference for probability of falls in favour of YOPD patients (OR 0.51, *p* = 0.029; Table 2).

**Table 2:** Summary of results over time for primary outcomes unadjusted

		Timeline*	YOPD	LOPD	*p* value
			Mean ± SD or *n* (%)	Mean ± SD or *n* (%)	
PDQ39 total		Baseline	19.9 ± 15.2	21.0 ± 14.3	0.299
		1-yr of follow-up	21.4 ± 15.4	22.1 ± 15.0	0.672
		2-yr of follow-up	22.1 ± 15.5	24.7 ± 15.8	0.165
		3-yr of follow-up	23.6 ± 17.6	25.8 ± 15.7	0.322
MCSI total		baseline	13.9 ± 13.8	16.0 ± 13.9	0.137
		1-yr of follow-up	14.2 ± 14.1	17.1 ± 14.6	0.201
		2-yr of follow-up	17.1 ± 17.2	19.2 ± 16.3	0.461
		3-yr of follow-up	14.2 ± 13.7	20.2 ± 13.9	**0.049**
Falls	None/rare	Baseline	225 (94.1%)	531 (90.3%)	0.074
	At least Monthly		14 (5.9%)	57 (9.7%)	
	None/rare	1-yr of follow-up	127 (91.4%)	252 (85.4%)	0.083
	At least Monthly		12 (8.6%)	43 (14.6%)	
	None/rare	2-yr of follow-up	96 (96.0%)	208 (85.2%)	0.005
	At least Monthly		4 (4.0%)	36 (14.8%)	
	None/rare	3-yr of follow-up	77 (96.3%)	141 (84.9%)	0.009
	At least Monthly		3 (3.8%)	25 (15.1%)	
Immediate five-word recall		Baseline	0.5 ± 0.5	− 0.2 ± 1.1	** < 0.0001**
		1-yr of follow-up	0.4 ± 0.6	− 0.1 ± 1.0	** < 0.0001**
		2-yr of follow-up	0.4 ± 0.6	− 0.3 ± 1.2	** < 0.0001**
		3-yr of follow-up	0.5 ± 0.6	− 0.2 ± 1.1	** < 0.0001**
Verbal fluency		Baseline	0.7 ± 1.0	− 0.3 ± 0.9	** < 0.0001**
		1-yr of follow-up	0.8 ± 1.0	− 0.3 ± 0.9	** < 0.0001**
		2-yr of follow-up	0.8 ± 1.1	− 0.4 ± 0.9	** < 0.0001**
		3-yr of follow-up	0.7 ± 1.0	− 0.4 ± 0.9	** < 0.0001**
Delayed five-word recall		Baseline	0.5 ± 0.8	− 0.2 ± 1.0	** < 0.0001**
		1-yr of follow-up	0.7 ± 0.7	− 0.2 ± 1.0	** < 0.0001**
		2-yr of follow-up	0.6 ± 0.7	− 0.3 ± 1.0	** < 0.0001**
		3-yr of follow-up	0.7 ± 0.9	− 0.1 ± 1.0	** < 0.0001**

*YOPD* young-onset Parkinson’s disease, *LOPD* late-onset Parkinson’s disease *PDQ-39* Parkinson’s disease questionnaire 39. *MCSI* modified caregiver strain index

Values stated as mean +  − standard deviation or *n* (%)

The bold values indicate p < 0.05

### Longitudinal results

Analyses unadjusted for covariates showed no group differences for PDQ-39, MCSI and falls, except for MCSI score after 3 years of follow-up. Differences for standardized cognitive tests showed significant differences at follow-up after 1, 2 and 3 years after baseline (*p* < 0.0001; Table 3).

**Table 3:** Summary of results over time for primary outcomes adjusted for disease duration

	Coëfficiënt	Standard error	P value	odds ratio
PDQ-39 total				
Baseline	− 0.71	1.03	0.492	
1 yr of follow-up	− 2.26	1.01	**0.025**	
2 yr of follow-up	− 3.82	1.19	**0.001**	
3 yr of follow-up	− 5.37	1.50	** < 0.001**	
MCSI total				
Baseline	− 1.788	1.447	0.217	
1 yr of follow-up	− 3.579	1.403	**0.011**	
2 yr of follow-up	− 5.370	1.751	**0.002**	
3 yr of follow-up	− 7.161	2.322	**0.002**	
Falls (probability of having fall at least monthly)				
Baseline	− 0.67	0.31	**0.029**	0.51
1 yr of follow-up	− 1.02	0.25	** < 0.0001**	0.36
2 yr of follow-up	− 1.37	0.39	** < 0.001**	0.25
3 yr of follow-up	− 1.72	0.60	**0.004**	0.18
Cognition_Immediate five-word recall standardized				
Baseline	0.70	0.07	** < 0.0001**	
1 yr of follow-up	0.75	0.07	** < 0.0001**	
2 yr of follow-up	0.80	0.09	** < 0.0001**	
3 yr of follow-up	0.84	0.12	** < 0.0001**	
Cognition_verbal standardized				
Baseline	1.03	0.06	** < 0.0001**	
1 yr of follow-up	1.10	0.05	** < 0.0001**	
2 yr of follow-up	1.18	0.08	** < 0.0001**	
3 yr of follow-up	1.25	0.12	** < 0.0001**	
Cognition delayed five-word recall standardized				
Baseline	0.70	0.07	** < 0.0001**	
1 yr of follow-up	0.78	0.06	** < 0.0001**	
2 yr of follow-up	0.86	0.09	** < 0.0001**	
3 yr of follow-up	0.94	0.12	** < 0.0001**	

Number of comorbidities and number of medications before baseline

*PDQ-39* Parkinson’s disease questionnaire 39; *MCSI* modified caregiver strain index

The bold values indicate p < 0.05

After adjusting for covariates, PDQ-39 scores were significantly lower for YOPD patients at all three follow-up moments. PDQ-39 score differences between groups worsened from − 2.26 (*p* = 0.025) after 1 year of follow-up to − 5.37 (*p* < 0.001) after 3 years of follow-up. The same can be seen for the results for MCSI scores (Table 2). Differences in MCSI score increased from − 3.579 (*p* = 0.011) after 1 year of follow-up to − 7.161 (*p* = 0.002) after 3 years of follow-up.

**Fig. 2:**
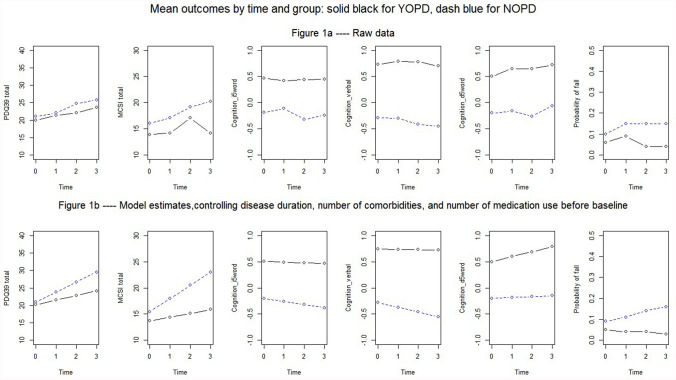
Mean outcomes by time and group, adjusted and unadjusted results over time in years. *PDQ39* Parkinson’s Disease questionnaire 39

The number of falls increased in both groups over time, with the YOPD group having a lower risk of falls during follow-up (OR 0.51–0.18, *p* < 0.05). YOPD patients performed better for all three cognitive tests over time, with increasing mean differences for standardized cognition scores (Fig. 2).

## Discussion

YOPD patients had better PDQ-39, better MCSI scores, fewer falls and better cognitive functioning after the first visit, with increasing differences between groups over time, supporting our hypothesis of YOPD patients having better quality of life and less caregiver strain.

Our findings extend similar experiences with other diseases (breast cancer and endometriosis) that also have a high impact on quality of life [10, 11, 15]. Earlier studies into age-related differences for PDQ-39 scores in PD patients showed a negative impact of earlier onset on quality of life [8, 9]. It is postulated that this is due to a variety of factors including marital conflicts, difficulties with family live, social isolation and loss of occupation [16]. However, these studies used smaller groups for young-onset PD patients with partially self-referred patients, as well adjusting for different confounders and were often cross-sectional. When compared to the general population, there is evidence that YOPD patients have greater worsening of quality of life mostly due to physical limitations and role expectation [17].

Comorbidities and medication use are important influencing factors of quality of life [18, 19]. In line with other studies, LOPD patients had more comorbidities compared to YOPD patients, possibly leading to confounding. Most importantly, we have performed multivariate analyses, adjusting for comorbidities and medication use. The difference between symptomatic and asymptomatic comorbidities can be subtle. To reduce bias, all comorbidities were assessed by the researcher, and discussed with the treating physician if needed.

Caregiver strain is not well documented in relation to YOPD. Increased caregiver burden is associated with patients’ advancing age [20, 21]. Our results support this relation between age of onset and caregiver strain, with older patients having a higher burden compared to YOPD patients. Also, earlier studies suggested that quality of life of patients and caregiver strain are strongly correlated to each other, MCSI and PDQ39 scores showing similar trends over time. Although the literature has little good-quality studies, we can conclude that caregiver strain is increased in people confronted with PD. There is some evidence for interventions to reduce caregiver strain which include education for the person with PD and the caregiver, psychotherapy targeting psychiatric symptoms in the caregiver and management of neuropsychiatric symptoms in the person living with PD [22]. Lastly, caregiver strain is influenced by cultural perceptions of disease and caregiving, leading to different results in other parts of the world [23, 24].

Our study suggests that YOPD patients experience fewer falls from baseline to the end of follow-up, similar to other large studies into predictors of falls in PD [12, 25]. A recent study using data from the PF QII-study reported no effect of age on falls. However, since we did not adjust for disease variables like HY-stage, our outcomes could be explained by differences in disease characteristics of the YOPD group, more closely resembling the general YOPD population. Fall diaries, electronically or paper based are the golden standard to measure falls [26], but impose a significant burden for patients. This study refrained from use of these instruments, possibly leading to underestimation of the number of falls. Nevertheless, the dichotomous outcome categorizing falls in ‘none/rarely’ and ‘at least monthly’, likely decreasing recall bias and subsequent underestimation of the number of falls.

The Parkinson’s Foundation QII-study is the largest international prospective study into quality of life of patients with PD. Earlier studies have shown a significant impact of the diagnosis of PD on patients and their caregivers with a difference between YOPD and LOPD, but only cross-sectionally [27]. This is the first study demonstrating longitudinal differences in quality of life, caregiver strain and falls. There are limitations of this study. There is a significant loss to follow-up for the longitudinal analysis, leading to possible attrition bias. We have analysed data cross-sectionally as well as longitudinally, for a relatively short duration of 3 years. The MCSI has not been validated for measuring caregiver strain among PD patients. However, many other studies demonstrated its reliability and validity for use in an elderly population [28]. The QII questionnaire does not report on genetic substrates of PD, or more extensively on motor symptoms (with for example the UPDRS-m) or the effects of caregiver strain. Nonetheless, we did report multiple on multiple subgroups of the latter, including physical strain (supplementary table 1.). Lastly, we did not report on employment status for the same reason. We feel our results support more extensive research into this topic.

In conclusion, this study described that YOPD patients are a group with a significantly better quality of life and slower disease progression over time compared to LOPD patients. This offers some consolation for the future perspectives for YOPD patients and creates the possibility of bringing a positive message for these patients in daily clinical practice.

## Electronic supplementary material

Below is the link to the electronic supplementary material.

Supplementary file1 (DOCX 45 kb)
