# Supplementary material for: Better quality of life and less caregiver strain in young-onset Parkinson’s disease: a multicentre retrospective cohort study
Source: J Neurol. 2020 Oct 27;268(3):1102–9. doi: 10.1007/s00415-020-10266-y (PMC7914232; doi:10.1007/s00415-020-10266-y)
Supplement: Supplementary file 1 — Supplementary file1 (DOCX 45 kb) [file 415_2020_10266_MOESM1_ESM.docx]

Supplementary table 1. Sub-scores of PDQ39 and MCSI, symptomatic comorbities and medication use. PDQ39: Parkinson’s Disease Questionnaire-39. MCSI: Modified Caregiver Strain Index. ADL: activities of daily living.

|  |  | Total | YOPD | LOPD | **p-value** |
| --- | --- | --- | --- | --- | --- |
| **PDQ-39** | | | | | |
| **PDQ39 whole** | Patient | 847 | 267 (98.2%) | 580 (84.7%) | **<0.0001** |
|  | Patient & caregiver | 100 | 4 (1.5%) | 96 (14.0%) |  |
|  | Caregiver | 10 | 1 (0.4%) | 9 (1.3%) |  |
| PDQ39 Mobility |  | 10.8±10.6 | 6.7±7.6 | 12.4±11.2 | **<.0001** |
| PDQ39 ADL |  | 6.1±5.6 | 5.1±5.0 | 6.5±5.8 | **<0.001** |
| PDQ39 Emotion |  | 5.3±4.5 | 5.8±4.6 | 5.1±4.5 | **0,050** |
| PDQ39 Stigma |  | 2.5±3.2 | 3.8±3.4 | 2.0±3.0 | **<.0001** |
| PDQ39 Social Support |  | 1.0±1.7 | 1.3±2.2 | 0.9±1.5 | **0,001** |
| PDQ39 Cognition |  | 3.6±3.0 | 2.9±3.1 | 3.9±3.0 | **<.0001** |
| PDQ39 Communication |  | 1.9±2.2 | 1.9±2.2 | 2.0±2.1 | 0,508 |
| PDQ39 Pain |  | 3.3±2.6 | 3.4±2.7 | 3.3±2.6 | 0,444 |
| PDQ39 total |  | 20.7±14.6 | 19.9±15.2 | 21.0±14.3 | 0,299 |
| **MCSI** | | | | |  |
| Physical Strain |  | 1.7±2.2 | 1.4±2.1 | 1.8±2.2 | 0,113 |
| Social Constraints |  | 3.5±3.4 | 2.5±2.9 | 3.8±3.5 | **<0.001** |
| Financial Strain |  | 0.5±1.1 | 0.9±1.4 | 0.4±0.9 | **<.0001** |
| Time Constraints |  | 2.3±2.2 | 2.2±2.3 | 2.4±2.2 | 0,450 |
| Interpersonal Strain |  | 2.1±2.7 | 2.2±2.5 | 2.1±2.8 | 0,8 |
| Elder Demanding / Manipulative behaviour |  | 0.8±1.5 | 0.5±1.0 | 0.9±1.6 | **0,003** |
| MCSI total |  | 15.5±13.9 | 13.9±13.8 | 16.0±13.9 | 0,137 |
| **Symptomatic comorbidities** | | | | |  |
| Heart | No | 857 | 264 (97.1%) | 593 (86.1%) | **<0.0001** |
|  | Yes | 104 | 8 (2.9%) | 96 (13.9%) |  |
| Respiratory | No | 918 | 264 (97.1%) | 654 (94.9%) | 0,149 |
|  | Yes | 43 | 8 (2.9%) | 35 (5.1%) |  |
| Diabetes | No | 932 | 270 (99.3%) | 662 (96.1%) | **0,009** |
|  | Yes | 29 | 2 (0.7%) | 27 (3.9%) |  |
| Cancer | No | 909 | 270 (99.3%) | 639 (92.9%) | **<0.0001** |
|  | Yes | 51 | 2 (0.7%) | 49 (7.1%) |  |
| Arthritis | No | 773 | 259 (95.2%) | 514 (74.6%) | **<0.0001** |
|  | Yes | 188 | 13 (4.8%) | 175 (25.4%) |  |
| Other | No | 850 | 248 (93.2%) | 602 (90.7%) | 0,207 |
|  | Yes | 80 | 18 (6.8%) | 62 (9.3%) |  |
| Hospital admissions | No | 745 | 224 (98.7%) | 521 (93.5%) | 0,003 |
|  | Yes | 39 | 3 (1.3%) | 36 (6.5%) |  |
| ER visit | No | 742 | 221 (96.5%) | 521 (93.4%) | 0,085 |
|  | Yes | 45 | 8 (3.5%) | 37 (6.6%) |  |
| Injury | No | 923 | 267 (98.2%) | 656 (95.1%) | **0,029** |
|  | Yes | 39 | 5 (1.8%) | 34 (4.9%) |  |
| Infection | No | 949 | 269 (98.9%) | 680 (98.6%) | 0,675 |
|  | Yes | 13 | 3 (1.1%) | 10 (1.4%) |  |
| Pneumonia | No | 954 | 272 (100.0%) | 682 (98.8%) | 0,075 |
|  | Yes | 8 |  | 8 (1.2%) |  |
| Trauma | No | 961 | 272 (100.0%) | 689 (99.9%) | 0,530 |
|  | Yes | 1 |  | 1 (0.1%) |  |
| **Medication use before first visit** | | | | |  |
| Any form levodopa | No | 240 | 135 (49.8%) | 105 (15.2%) | **<0.0001** |
|  | Yes | 720 | 136 (50.2%) | 584 (84.8%) |  |
| Dopamine agonist | No | 719 | 118 (43.7%) | 601 (87.2%) | **<0.0001** |
|  | Yes | 240 | 152 (56.3%) | 88 (12.8%) |  |
| MAO-B inhibitor | No | 755 | 171 (63.3%) | 584 (84.8%) | **<0.0001** |
|  | Yes | 204 | 99 (36.7%) | 105 (15.2%) |  |
| COMT inhibitor | No | 911 | 252 (93.7%) | 659 (95.6%) | 0,206 |
|  | Yes | 47 | 17 (6.3%) | 30 (4.4%) |  |
| Amantadine | No | 889 | 233 (86.3%) | 656 (95.2%) | **<0.0001** |
|  | Yes | 70 | 37 (13.7%) | 33 (4.8%) |  |
| Antidepressant | No | 737 | 218 (81.0%) | 519 (75.3%) | 0,059 |
|  | Yes | 221 | 51 (19.0%) | 170 (24.7%) |  |
| Cognitive enhancers | No | 898 | 265 (98.5%) | 633 (91.9%) | **<0.001** |
|  | Yes | 60 | 4 (1.5%) | 56 (8.1%) |  |
| Antipsychotics | No | 931 | 263 (97.8%) | 668 (97.1%) | 0,563 |
|  | Yes | 26 | 6 (2.2%) | 20 (2.9%) |  |
| Anticholinergics | No | 905 | 250 (92.9%) | 655 (95.2%) | 0,164 |
|  | Yes | 52 | 19 (7.1%) | 33 (4.8%) |  |
| **Medication use after first visit** | | | | |  |
| Any form levodopa | No | 207 | 128 (47.6%) | 79 (11.5%) | **<0.0001** |
|  | Yes | 748 | 141 (52.4%) | 607 (88.5%) |  |
| Dopamine agonist | No | 698 | 103 (38.3%) | 595 (86.7%) | **<0.0001** |
|  | Yes | 257 | 166 (61.7%) | 91 (13.3%) |  |
| MAO-B inhibitor | No | 734 | 156 (58.0%) | 578 (84.3%) | **<0.0001** |
|  | Yes | 221 | 113 (42.0%) | 108 (15.7%) |  |
| COMT inhibitor | No | 899 | 247 (91.8%) | 652 (95.0%) | 0,057 |
|  | Yes | 56 | 22 (8.2%) | 34 (5.0%) |  |
| Amantadine | No | 875 | 225 (83.6%) | 650 (94.8%) | **<0.0001** |
|  | Yes | 80 | 44 (16.4%) | 36 (5.2%) |  |
| Antidepressant | No | 722 | 213 (79.2%) | 509 (74.3%) | 0,114 |
| Cognitive enhancers | No | 890 | 265 (98.5%) | 625 (91.0%) | **<0.0001** |
|  | Yes | 66 | 4 (1.5%) | 62 (9.0%) |  |
| Antipsychotics | No | 929 | 263 (97.8%) | 666 (97.2%) | 0,637 |
|  | Yes | 25 | 6 (2.2%) | 19 (2.8%) |  |
| Anticholinergics | No | 897 | 250 (92.9%) | 647 (94.7%) | 0,286 |
| **Other treatments before first visit** | | | | | |
| Physical therapy | No | 587 | 202 (74.3%) | 385 (55.8%) | **<0.0001** |
|  | Yes | 375 | 70 (25.7%) | 305 (44.2%) |  |
| Occupational therapy | No | 831 | 251 (92.3%) | 580 (84.1%) | **0,001** |
|  | Yes | 131 | 21 (7.7%) | 110 (15.9%) |  |
| Speech therapy | No | 863 | 257 (94.5%) | 606 (87.8%) | **0,002** |
|  | Yes | 99 | 15 (5.5%) | 84 (12.2%) |  |
| Exercise program | No | 306 | 87 (32.0%) | 219 (31.8%) | 0,952 |
|  | Yes | 655 | 185 (68.0%) | 470 (68.2%) |  |
| Social worker/counseling | No | 867 | 243 (89.3%) | 624 (90.4%) | 0,608 |
|  | Yes | 95 | 29 (10.7%) | 66 (9.6%) |  |
| Mental Health therapy or referral | No | 891 | 247 (90.8%) | 644 (93.3%) | 0,177 |
|  | Yes | 71 | 25 (9.2%) | 46 (6.7%) |  |
| Deep brain stimulation | No | 954 | 266 (97.8%) | 688 (99.7%) | **0,003** |
|  | Yes | 8 | 6 (2.2%) | 2 (0.3%) |  |
| **Other treatments after first visit** | | | | | |
| Physical therapy | No | 678 | 222 (81.6%) | 456 (66.6%) | **<0.0001** |
|  | Yes | 279 | 50 (18.4%) | 229 (33.4%) |  |
| Occupational therapy | No | 894 | 257 (94.5%) | 637 (92.9%) | 0,363 |
|  | Yes | 64 | 15 (5.5%) | 49 (7.1%) |  |
| Speech therapy | No | 870 | 257 (94.5%) | 613 (89.4%) | **0,013** |
|  | Yes | 88 | 15 (5.5%) | 73 (10.6%) |  |
| Exercise program | No | 261 | 75 (27.7%) | 186 (27.2%) | 0,880 |
|  | Yes | 694 | 196 (72.3%) | 498 (72.8%) |  |
| Social worker/counseling | No | 902 | 248 (91.9%) | 654 (95.3%) | **0,036** |
|  | Yes | 54 | 22 (8.1%) | 32 (4.7%) |  |
| Mental Health therapy or referral | No | 890 | 246 (91.1%) | 644 (93.9%) | 0,129 |
|  | Yes | 66 | 24 (8.9%) | 42 (6.1%) |  |
| Deep brain stimulation | No change | 925 | 260 (97.7%) | 665 (99.6%) | **0,011** |
|  | Refer for evaluation | 9 | 6 (2.3%) | 3 (0.4%) |  |
